# The current situation of canine dirofilariosis in Hungary

**DOI:** 10.1007/s00436-019-06478-5

**Published:** 2019-11-21

**Authors:** Róbert Farkas, Viktória Mag, Mónika Gyurkovszky, Nóra Takács, Károly Vörös, Norbert Solymosi

**Affiliations:** 1 Department of Parasitology and Zoology, University of Veterinary Medicine, István u. 2, Budapest, H-1078 Hungary; 2 Department and Clinic of Internal Medicine, University of Veterinary Medicine, István u. 2, Budapest, H-1078 Hungary; 3 Centre for Bioinformatics, University of Veterinary Medicine, István u. 2, Budapest, H-1078 Hungary

**Keywords:** *Dirofilaria immitis*, *repens*, Hungary, Spreading, Spatial pattern, Environmental association

## Abstract

Between April and September 2017, blood samples were collected from 344 randomly selected dogs older than 1 year in 180 settlements of 19 counties in Hungary. The dogs lived exclusively outdoors, had never travelled and had neither been examined for *Dirofilaria* infection nor treated against mosquitoes with insecticides or/and filarioid worms with macrocyclic lactones. *Dirofilaria* infection was examined with a modified Knott’s test for microfilariae, DiroCHEK®, for the presence of *D. immitis* antigen, as well as by multiplex and conventional PCR. Altogether, 77 (22.4%) dogs living in 58 settlements of 17 counties were found to be infected with one or both *Dirofilaria* species based on the PCR techniques. Twenty-eight (8.1%) and 38 (11.1%) dogs were infected with *D. immitis* and *D. repens*, respectively. Coinfections were recorded in 11 samples (3.2%) collected in 11 locations of 8 counties. The results confirmed that both dirofilarioses are endemic in dogs and the eastern areas of the country are hyperendemic for heartworm disease. Temperature showed a significant association with the prevalence of *D. immitis* (OR 2.41, 95% CI 1.24–4.86, *p* = 0.012) but not with that of *D. repens* (OR 1.37, 95% CI 0.78–2.47, *p* = 0.286). The prevalence of neither *D. immitis* (OR 0.99, 95% CI 0.98–1.00, *p* = 0.213) nor *D. repens* (OR 1.01, 95% CI 0.99–1.01, *p* = 0.094) showed a significant correlation with precipitation. The number of yearly growing degree days (GDD) based on the lifecycle of *Dirofilaria* in mosquitoes ranged between 3.73 and 7.57 for the Hungarian districts. The GDD showed a significant positive association with the prevalence of *D. immitis* (OR 2.38, 95% CI 1.43–4.15, *p* = 0.001) and a non-significant positive relationship with that of *D. repens* (OR 1.25, 95% CI 0.83–1.95, *p* = 0.291).

## Introduction

One of the most pathogenic parasites of dogs, *Dirofilaria immitis*, can cause life-threatening heartworm disease (other name cardiopulmonary dirofilariosis) worldwide (Simón et al. 2012). *Dirofilaria repens*, the causative agent of subcutaneous dirofilariosis of domestic dogs, occurs in Europe, Asia and Africa only. Both species can infect several other mammalian species, especially wild canids, such as red foxes, golden jackals, wolves, ferrets and rarely cats (Otranto and Deplazes 2019). In addition, they are zoonotic parasites: *D. repens* causes ocular/subcutaneous while *D. immitis* benign pulmonary dirofilariosis in humans (McCall et al. 2008; Simón et al. 2012; Morchón et al. 2012; Capelli et al. 2018). The infective larvae of these filarioid helminths (Spirurida: Onchocercidae) develop in mosquitoes. About 60–70 species of the family Culicidae belonging to several genera are regarded as potential intermediate hosts and vectors around the world; however, their vector competence has been proven only in a few cases (McCall et al. 2008).

Until the last decade of the twentieth century, both parasites had occurred mainly in the southern European countries such as Italy, Portugal, Spain, France and Greece, where dirofilariosis was considered to be present historically (Trotz-Williams and Trees 2003; Genchi et al. 2005; Morchón et al. 2012). Recent epidemiological studies have confirmed that both helminths have appeared and become endemic in many countries of Central and Eastern Europe where they have caused an increasing number of autochthonous infections among the local dogs (Morchón et al. 2012; Tasić-Otašević et al. 2015; Capelli et al. 2018). The quick geographical spreading of these parasites to European areas where they were not known to be present previously can be explained by several factors. The importance of the increasing length of warm weather periods due to the climate change has to be underlined as one of the major determinants of the development, activity and seasonal survival of mosquitoes as well as of the development of *Dirofilaria* larvae in the vectors (Medlock et al. 2007; Genchi et al. 2009, 2011). The introduction of the Pet Travel Scheme in 2000 contributed to the spread of dirofilariosis by allowing easier movement of infected, microfilaraemic dogs across Europe from endemic areas (Genchi et al. 2009, 2011).

The first autochthonous *D. repens* infections of dogs were reported at the end of the 1990s in Hungary (Fok et al. 1998; Széll et al. 1999). During the first nationwide epidemiological surveys, the mean prevalence of *D. repens* was 14% in microfilaraemic dogs (Fok et al. 2007). Heartworm infection was diagnosed in dogs previously living in the USA (Boros et al. 1982; Vörös et al. 2000). Jacsó et al. (2009) reported the first autochthonous *D. immitis* infection of a local dog which lived in the eastern part of the country. Since that time, the number of confirmed heartworm infections has increased (Farkas et al. 2014; Túri and Hetyey 2014; Bacsadi et al. 2016; Trájer et al. 2016; Vörös et al. 2017; Bagi et al. 2017) and worms were found during the necropsy of a ferret (Molnár et al. 2010), some red foxes and golden jackals (Tolnai et al. 2014).

One of the objectives of this study was to get up-to-date information about the geographical distribution of *Dirofilaria*-infected dogs in the country. The association between the prevalence and the climatic conditions was also analysed to describe the relationships quantitatively.

## Materials and methods

### Study areas, animals and sampling

**Fig. 1:**
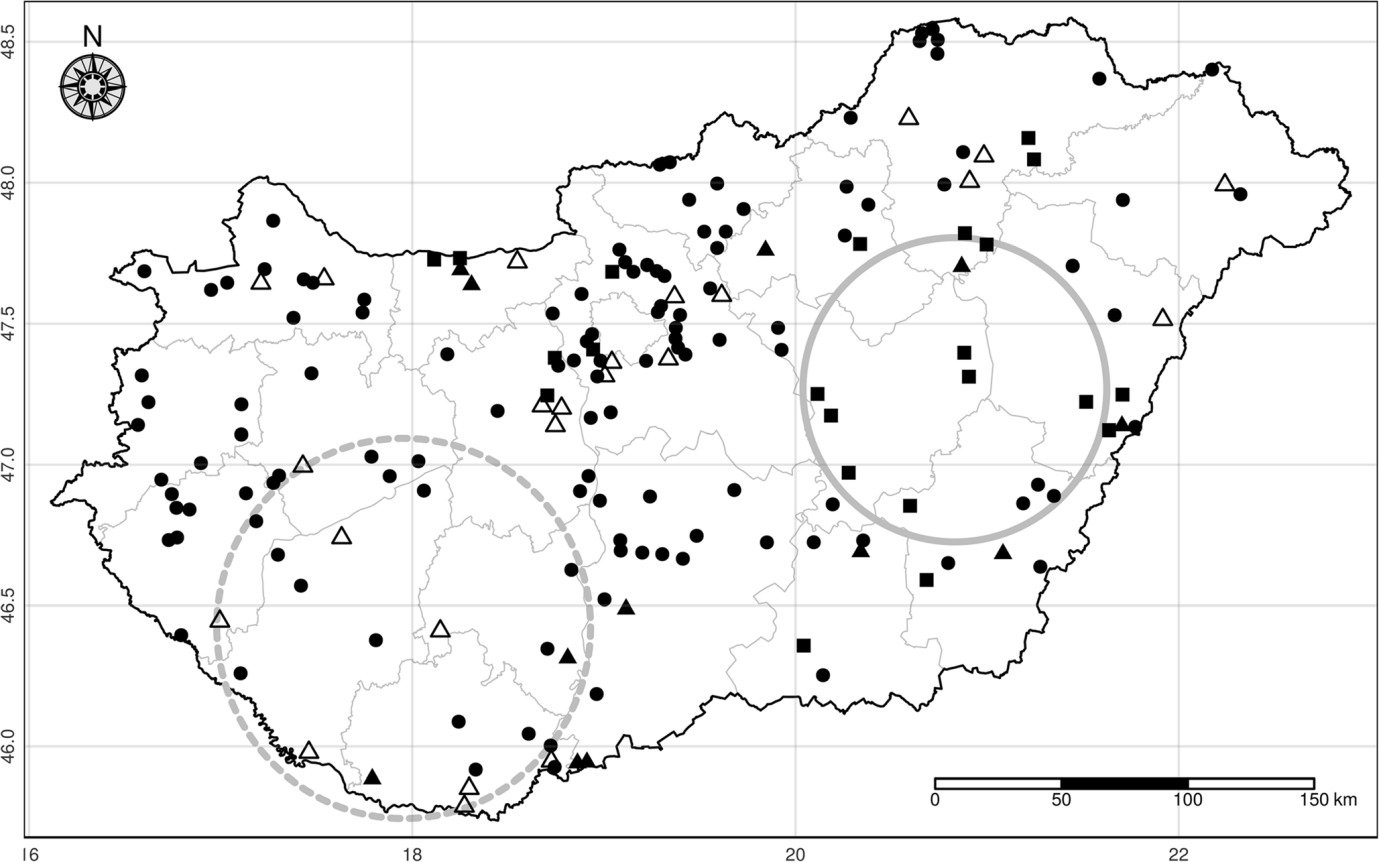
Sampling locations of the survey. Settlements with no infected dogs are indicated by black dots, while places of infection by *D. immitis* and *D. repens* and by both species are represented by filled boxes, empty triangles and filled triangles, respectively. Detected spatial cluster for *D. immitis* (solid line) and *D repens* (dashed line)

Between April and September 2017, blood samples were collected from 344 randomly selected dogs older than 1 year in 180 settlements of all the 19 counties in Hungary, with the exception of the capital, Budapest (Fig. 1). The number of examined dogs per county was calculated based on the number of animals vaccinated against rabies in 2016. Most of the animals were medium-sized mixed breed dogs, aged between 1 and 13 years. They lived exclusively outdoors, had never travelled out of their county and had neither been examined for *Dirofilaria* infection nor treated against mosquitoes with insecticides and filarioid worms with macrocyclic lactones. With the owners’ consent, a 2-mL blood sample was withdrawn from the cephalic vein of each dog using two labelled tubes without anticoagulant and with EDTA. After centrifugation, the serum samples were stored at − 20 °C until further processing.

### Examination for microfilariae

The presence of circulating microfilariae was examined using a modified Knott’s test (Genchi et al. 2007).

### Serologic assay

Serum samples were tested for the presence of *D. immitis* antigen produced by adult female heartworms with DiroCHEK® (Synbiotics Corporation, San Diego, USA), in accordance with the manufacturer’s instructions.

### Molecular identification

For a specific diagnosis, DNA was isolated from 0.2 mL of blood from each sample containing microfilariae based on the modified Knott’s test using NucleoSpin® Tissue kit (Macherey-Nagel GmbH, Düren, Germany). Multiplex and conventional PCR reactions targeting fragments of 12S rDNA of both *Dirofilaria* spp. and 16S rRNA of *D. immitis* were used, respectively (Liu et al. 2005; Gioia et al. 2010). In each reaction set, a positive control (DNA extracted from adult nematodes) and a sample with no DNA were included. The PCR products were visualised by gel electrophoresis, and their molecular weight was assessed by comparison to a molecular marker (PCRBIO Ladder IV 100–1500 bp DNA Ladder, PCR Biosystems Ltd., London, UK).

### Spatial and climate association analyses

The prevalence of dirofilariosis was estimated based on the aggregated samples at NUTS 4 level. The spatial clustering was tested by Kulldorff spatial cluster detection method using Poisson likelihood with 50% as the maximum window size (Kulldorff and Nagarwalla 1995). Environmental associations were analysed by logistic regression. As independent variables, climate data aggregated on level NUTS 4 were applied (Gelman and Hill 2006). The climate variables were calculated using the 2-m temperature and yearly total precipitation obtained with 0.125° spatial resolution from the ECMWF ERA-Interim daily repository (Dee et al. 2011) for the period 2008–2017. Besides the raw temperature data, the number of *Dirofilaria* generations based on the growing degree days (GDD) was calculated following the method described by Genchi et al. (2005). For temperature, GDD and precipitation, the yearly average was included in the models. All statistical analyses and visualisation were performed in R (R Core Team 2018).

## Results

**Fig. 2:**
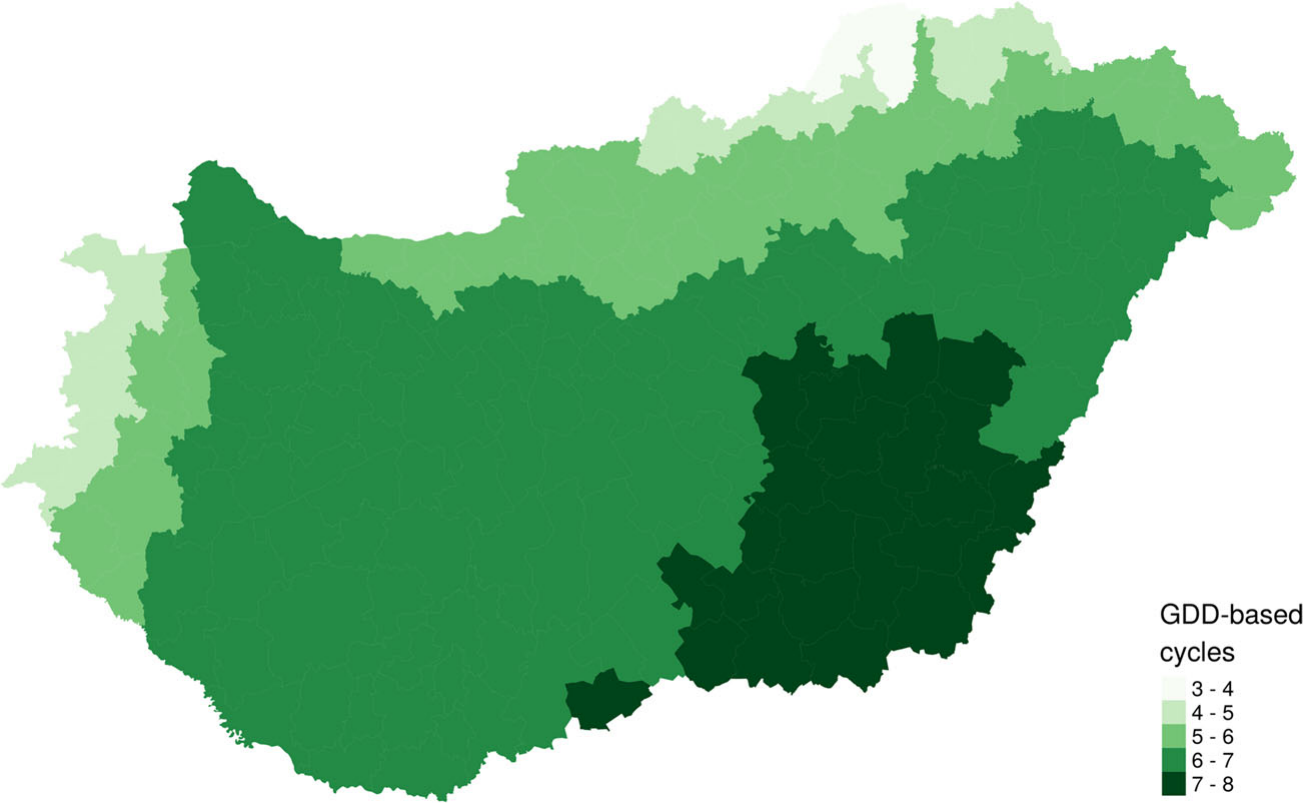
The growing degree days (GDD)-based yearly mean lifecycles of *Dirofilaria* calculated on NUTS 4 level in Hungary from ECMWF ERA Interim daily data for the period 2008–2017

Altogether, 77 out of the 344 dogs from 58 settlements of 17 counties were found to be infected by one or both *Dirofilaria* species based on the serological and PCR results (Fig. 1). The overall prevalence of dirofilariosis was 22.4% (95% CI 18.30–27.08). Twenty-eight (8.1%) and 38 (11.1%) dogs were infected with *D. immitis* and *D. repens*, respectively. No occult dirofilariosis occurred. Coinfections were recorded in 11 (3.2%) samples collected in 11 locations of 8 counties (Fig. 1). The total number of animals with heartworms was 39 (11.3%): these dogs lived in 34 settlements of 12 counties. The prevalence of *D. repens* was slightly higher (49/344, 14.2%); the infected dogs lived in 35 settlements of 16 counties (Fig. 1). By the Kulldorff scan method, a non-identical, significant cluster was identified for *D. immitis* (*p* < 0.001) and *D. repens* (*p* = 0.044) (Fig. 1). However, non-significant (*p* = 0.07) spatial aggregation was found for the pooled data of both species. Temperature showed a significant association with the prevalence of *D. immitis* (OR 2.41, 95% CI 1.24–4.86, *p* = 0.012) but not with that of *D. repens* (OR 1.37, 95% CI 0.78–2.47, *p* = 0.286). The prevalence of neither *D. immitis* (OR 0.99, 95% CI 0.98–1.00, *p* = 0.213) nor *D. repens* (OR 1.01, 95% CI 0.99–1.01, *p* = 0.094) showed a significant correlation with precipitation. The number of yearly GDD-based life cycles of *Dirofilaria* in vectors ranged between 3.73 and 7.57 for the Hungarian districts (Fig. 2). It showed a significant positive association with the prevalence of *D. immitis* (OR 2.38, 95% CI 1.43–4.15, *p* = 0.001) and a non-significant positive relationship with that of *D. repens* (OR 1.25, 95% CI 0.83–1.95, *p* = 0.291).

## Discussion

Although it was suspected that *D. repens* infections had occurred in Hungary between 1879 and 1951 (Kotlán 1951), the first confirmed autochthonous cases of dogs (Fok et al. 1998; Széll et al. 1999) and humans (Szénási et al. 2008) were reported at the end of the twentieth century and later. In the first nationwide epidemiological survey of 826 dogs, 116 (14.0%) were positive for *D. repens* microfilariae and more than half of them lived along the Danube and Tisza rivers with huge areas of mosquito breeding sites (Fok et al. 2007). A few years later, a higher prevalence (19.6%) of *D. repens* was found when blood samples from 2278 dogs were examined with the modified Knott method (Jacsó 2014). The result of this study is in line with the previous findings, as the prevalence of *D. repens* was 14.2%. Our results confirm that *D. repens* is widely distributed in the local dogs, presenting a continuous risk of human infection (Dóczi et al. 2015). Microfilaraemic dogs occurred in 16 counties, but a significant (*p* = 0.044) cluster was found only in a region where *D. repens* was the most frequent filarioid parasite in mosquito samples collected in a 3-year surveillance programme between 2011 and 2013 (Kemenes et al. 2015). Taking into account the previous report about *Dirofilaria* infections in the country (Kotlán 1951), we suspect that *D. repens* appeared in Hungary and become endemic earlier than the end of the twentieth century as others hypothesised (Genchi et al. 2011; Capelli et al. 2018).

From the beginning of the 2000s, the focus of scientific interest shifted to *D. immitis* that causes a severe and potentially fatal cardiopulmonary disease in dogs (Simón et al. 2012). According to previous Hungarian reports, heartworm infection had been diagnosed pathologically only in dogs imported from the USA until 2000 (Boros et al. 1982; Vörös et al. 2000). Following the first autochthonous canine heartworm case (Jacsó et al., 2009), a few dozens of infected dogs have been reported (Farkas et al. 2014; Túri and Hetyey 2014; Bacsadi et al. 2016; Trájer et al. 2016; Vörös et al. 2017; Bagi et al. 2017). All of them were considered autochthonous because these animals had been born in Hungary and had never left the country. The prevalence of *D. immitis* obtained in this study is higher (11.3%) than that found in earlier serological (Farkas et al. 2014) and retrospective surveys based on the necropsy records of 2622 dogs (Bacsadi et al., 2016), indicating that the number of infected dogs has been increasing in the country. Mixed *Dirofilaria* infections were detected in 11 (3.2%) dogs in our study. Trájer et al. (2016) also found 5 coinfected dogs in the southern part of Hungary where 18 dogs were infected with *D. immitis* and 12 out of 56 with *D. repens*. These findings show that there is no spatial segregation of the two filarioid species in the given areas. The dogs having both dirofilarioses can be infected by mosquitoes of the same or different species as reported from Italy (Genchi et al. 2009). In our study, a few dogs infected with *D. immitis* were found in some counties but there was only one significant (*p* = 0.0002) cluster in the eastern part of Hungary where the first autochthonous case was diagnosed. It can be stated that this area is hyperendemic for heartworm disease. We assume that more *D. immitis*-infected local dogs facilitated the spreading of this nematode species there than in the other part of the country. It cannot be excluded definitively that *D. immitis* had also been present in the country before the twenty-first century because no epidemiological surveys had been carried out and no reliable diagnostic methods were available earlier. However, it is more plausible that *D. immitis* has only recently been introduced to Hungary because neither microfilariae nor adult worms of *D. immitis* had been found in local dogs (Fok et al. 2007) and red foxes earlier (Sréter et al. 2003). It is not known yet why *D. repens* has higher prevalence than *D. immitis*. The dogs infected with this helminth are often asymptomatic, and no specific serological tests are available, while heartworm infections cause severe clinical disease (McCall et al. 2008). There are several in-clinic serological test kits for detecting the circulating antigens of female heartworms (Capelli et al. 2018). Although Genchi et al. (2005) assumed the same requirements for the development of both species, further research is needed to study the effect of temperature on *D. repens*.

In Hungary, the occurrence and spread of both filarioid species are not surprising because the local climate and the abundance of mosquito vectors offer suitable conditions for the development and transmission of these parasites. The question to be answered is how these nematodes arrived and spread in the country. A possibility is that they were introduced from a neighbouring country where they had been reported. In a long-term monitoring carried out in Slovakia between 2005 and 2015, *D. immitis* was detected in 10 dogs of which 9 had mixed infection, and some autochthonous cases were revealed in the Komárno district, close to the border of Hungary (Čabanová et al. 2015; Miterpáková et al. 2016). In Serbia, a high prevalence of both species had been found in dogs (Tasić et al. 2008, 2012; Krstić et al., 2017). There are some reports about the prevalence and distribution of *Dirofilaria* spp. from Romania, too. Depending on the methods and sampling areas, significant variations of single and mixed dirofilarioses of dogs have been reported (Mircean et al. 2012; Ionică et al. 2015). It might be possible that infected red foxes and/or golden jackals arrived from Romania (Ionică et al. 2017) or Serbia (Penezić et al. 2014) where *Dirofilaria* infection of these wild canids was detected, which underlines their role as reservoir hosts in the dissemination of these nematodes. Hungarian authors (Kemenesi et al. 2015) reported that golden jackals may also facilitate the persistence of *D. repens* and could naturally widen the distribution area of that parasite. Nevertheless, we do not think that the role of wild canids should be considered regarding the geographical distribution of dirofilariosis in Hungary because only a few red foxes and two golden jackals shot in 8 counties were found to be infected, having a low number of heartworms without microfilaraemia (Tolnai et al. 2014). Marconcini et al. (1996) also found low numbers of adult heartworms in foxes without microfilaraemia. It is more plausible that the infection of the local wild canids may have originated from infected dogs inhabiting the same areas. We also hypothesise that local mosquitoes could be infected with microfilariae of *Dirofilaria* spp. originally from dogs infected abroad like those which arrived in Hungary with their owners for hunting from endemic countries. Stray dogs and dogs adopted from shelters pose a high risk in the epidemiology of both dirofilarioses because they are unlikely to receive proper examination and prevention. The likelihood of other carnivores such as cats or ferrets being involved in the transmission of *Dirofilaria* spp. should not be considered because microfilaraemia is absent or short-lasting in these hosts (Campbell and Blair 1978; McCall et al. 2008; Bajer et al. 2016). The chance that *Dirofilaria* spp. were introduced to Hungary with infected mosquitoes is very small, because the movement and ranges of these insects are limited (Genchi et al. 2005; Tsuda et al. 2008; Zittra et al. 2015). However, the importance of mosquitoes carried by the wind and humans in the geographical distribution of vector-borne pathogens cannot be excluded. To date, *Dirofilaria* infection in the local mosquitoes is scarcely known. Among 50 mosquito species known to be present in the country (Kenyeres and Tóth 2012), the specimens of the *Culex pipiens* complex seem to be important vectors of *Dirofilaria* spp. (Zittra et al. 2015) like in other European countries (Cancrini et al. 2006, Morchón et al. 2007; Čabanová et al. 2018). Some specimens of other mosquito species such as *Cx. modestus* and *Ocreatus caspius* infected with *Dirofilaria* larvae were caught in Southern Hungary. The potential involvement of *Oc. sticticus* mosquitoes in the natural transmission cycle of the parasites was also reported from the country (Kemenesi et al. 2015; Zittra et al. 2015). Taking into account the two main preconditions of the transmission of *Dirofilaria* spp. (Simón et al. 2012), we hypothesize that the presence of a minimum number of dogs infected with adult worms producing microfilariae facilitated establishment of both parasite species in the country.

More than a decade ago, Genchi et al. (2005) used temperature records obtained from all over Europe from 1977 to 1991 to predict the likely number of *Dirofilaria* generations in Europe. They reported that the yearly average predicted number of heartworm generations for Hungary was between 1 and 5. Following their method, we calculated the number of yearly growing degree days (GDD) using temperature data for the period 2008 through 2017. We obtained a higher estimate for the number of *Dirofilaria* generations per year. This can be explained by the increasing temperature due to global warming. Another possible reason for the differences might be the distinct sources of the applied meteorological datasets. The risk period for *Dirofilaria* transmission is seasonal in Hungary, with peaks in the summer, when the temperature is especially sufficient to facilitate the extrinsic incubation of *Dirofilaria* in vectors. We are aware of the fact that environmental temperature along with adequate moisture is one of the key factors in the epidemiology of dirofilariosis as others have stated (Medlock et al. 2007; Genchi et al. 2009). However, the movement of unprotected dogs against mosquitoes and dirofilariosis and other factors such as the population density of vectors and hosts, the potential mosquito-breeding habitats and the veterinarians’ awareness of the infection also play a critical role in the geographical distribution of both filarioid species.

## Conclusions

Both dirofilarioses are endemic in Hungary, and heartworm disease is more prevalent in the eastern areas of the country. This study further confirms the significant circulation of these filarioid parasites in the local domestic dogs. We assume that the administration of broad-spectrum chemoprophylactic products with endoparasiticidal and/or ectoparasiticidal activity for 8–12 months each year would assist in preventing these pathogenic and zoonotic parasitic infections. Further systematic monitoring studies are required to better understand the environmental risk factors and to identify the competent mosquito vectors in the epidemiology of local dirofilariosis. Particular attention should be paid to stray and shelter dogs.
